# Life style and longevity among initially healthy middle-aged men: prospective cohort study

**DOI:** 10.1186/1471-2458-13-831

**Published:** 2013-09-11

**Authors:** Trond Heir, Jan Erikssen, Leiv Sandvik

**Affiliations:** 1 Oslo Ischemia Study, Oslo University Hospital, Kirkeveien 166, N-0407, Oslo, Norway

**Keywords:** Prospective, Healthy men, Smoking, Overweight, Fitness, Longevity

## Abstract

**Background:**

Few studies have examined how various lifestyle factors in midlife predict longevity, and none of these studies have examined the impact of physical fitness. The present study aimed to examine longevity in relation to smoking, overweight and physical fitness.

**Methods:**

We prospectively studied longevity (defined as reaching at least 85 years of age) in relation to smoking status, body mass index and physical fitness in 821 healthy men between 51 and 59 years of age. Of these, 369 were smokers, 320 were overweight, and 31 were obese. The associations were adjusted for age, systolic blood pressure and cholesterol level, using multivariate logistic regression analysis. Deaths were registered until the 31st of December, 2006. Physical fitness was measured as the total work performed in a maximal exercise tolerance bicycle test.

**Results:**

252 men survived to the age of 85 years (30.7%). Smoking status was significantly and independently related to longevity; 37.2% of the non-smokers survived to the age of 85, and 22.8% of the smokers. Among non-smokers, overweight and physical fitness were significantly and independently related to longevity after adjustment for age, blood pressure and cholesterol level, but not among smokers. Among non-smokers with high physical fitness, 48.8% reached the age of 85 years, compared to 27.9% among non-smokers with low physical fitness.

**Conclusion:**

Lifestyle variables appear to be strong and independent predictors of longevity in initially healthy middle-aged men. The probability of longevity may be a useful concept when informing the general public about the benefits of a healthy lifestyle.

## Background

Risk factors for mortality have been thoroughly examined in a large number of studies over the last three decades. Several prospective studies have provided evidence of how various lifestyle factors in midlife are linked to mortality in initially healthy subjects [1-3]. Thus, it is well known that smoking, physical inactivity, low physical fitness, overweight, and obesity are associated with increased mortality.

Life expectancy is increasing in most industrialized countries, as is the number of people reaching the age of 85, a group often designated as the “oldest old”. Only a few prospective studies have addressed how lifestyle predicts the probability of surviving to the age of 85 or higher, hereafter denoted as longevity. In a study including 5820 Japanese-American middle-aged men without cardiovascular diseases and diabetes, non-smoking, normal weight and high grip strength were independent predictors of longevity [4,5]. Cardiovascular fitness was not measured in this study. In another study including 2357 US male physicians with an average age of 72 years at baseline [6], only unadjusted results on potential predictors of longevity were presented. Significant predictors at baseline were non-smoking, normal weight, high exercise frequency, normal blood pressure, and lack of chronic diseases. Cardiovascular fitness was not measured in this study either, and more than half of the participants had hypertension or a chronic disease at baseline.

The present study aimed to examine the associations between lifestyle factors in midlife and the probability of reaching the age of 85. The study was based on a cohort of initially healthy middle-aged Norwegian men constituting the Oslo Ischemia Study [3,7,8].

## Methods

### Design

The study has a prospective cohort design with long term follow-up of mortality.

### Subjects

The participants were recruited from five companies in Oslo, Norway, between August 28, 1972 and March 26, 1975. They were all male, both white- and blue-collar workers that were considered to be representative of the healthy, working male population of Norway. Healthy men, aged 40 to 60 years, were recruited based on a thorough evaluation of their company health records, which included annual or biannual health examinations. Exclusion criteria were known or suspected coronary or other heart diseases, advanced pulmonary diseases, diabetes mellitus, hypertension treated with drugs, advanced renal disease, liver disease, cancer and miscellaneous other diseases including neurological, muscular or joint diseases which would interfere with a planned exercise test. Furthermore, nobody was allowed to take drugs regularly. In total, 2341 employees fulfilled these criteria and were subsequently invited to take part in the study. 2014 of them (86 percent) accepted to participate and completed the study protocol.

Information about mortality until December 31, 2006, was obtained from the mortality registry of Statistics Norway. This registry contains information on all recorded deaths of Norwegian citizens living in Norway at time of death since 1^st^ of January 1951. Participants not found in the mortality register by December 31, 2006, were traced through another registry at Statistics Norway, and found to live in Norway at this date. Thus, our mortality data was 100% complete by December 31, 2006.

Individuals born later than December 31, 1921, could not have reached the age of 85 by the year 2006, and were therefore not included in our study, remaining 821 study participants. Age at enrolment ranged from 51 to 59 years with a mean of 55.3 years (SD = 2.8).

The study was approved by the Norwegian Social Data Services and the South-East Norway Committee for Medical Research Ethics. Approval to carry out and publish the study was given, knowing that a written consent from participants had not been obtained.

### Measurements

The study protocol encompassed a comprehensive medical history, physical examination, a panel of blood tests (including a lipid profile), and a maximal exercise tolerance bicycle test [3], which was carried out at Rikshospitalet University Hospital, Oslo. Participants were encouraged to continue exercising until exhaustion. Work capacity was calculated as the sum of the work performed (in kJ) at each workload until the termination of the test. Physical fitness (in tertiles) was measured as work capacity divided by body weight, and adjusted for age using linear regression. Body mass index (BMI) was computed from measured height and weight (the weight in kilograms divided by the square of the height in meters). Participants were divided into normal weight (BMI < 25), overweight (BMI 25–29.9), and obesity (BMI ≥30). Resting blood pressure was measured after the participant had been in the supine position for five minutes. Cholesterol levels were determined by standardized methods, as reported elsewhere [9]. The participants were examined between 7:30 am and 10:30 am and had been abstaining from eating and smoking for the preceding 12 hours. The registrations of each baseline variable used in this study were 100% complete.

### Statistical analysis

An independent sample t-test was used to compare mean differences between smokers and non-smokers, whereas a chi-square test was used for the BMI categories. We used cross-tables and logistic regression analysis to investigate the associations between longevity (reaching the age of 85) and the independent baseline variables: age, body mass index, physical fitness, cholesterol level, systolic blood pressure, and daily number of cigarettes. Age, systolic blood pressure, and cholesterol level were modeled as continuous variables. BMI scores were categorized as normal (BMI < 25), overweight (BMI: 25–29.9) and obese (≥ 30). Physical fitness was categorized into tertiles. Daily number of cigarettes was recorded as 0, 1–9, and ≥10. Statistical analysis was performed separately for smokers and non-smokers.

The results obtained from the regression analysis are presented as odds ratios with p-values and 95% confidence intervals.

For each covariate in each multivariable logistic regression model, we checked collinearity by calculating the variance inflation factor (VIF) statistic [10]. All VIF values were below 1.5, indicating that the models are not invalidated by collinearity.

In both the univariate and multivariate regression analysis we confirmed that the logits of the continuous variables were linear. For each multivariable model, interactions and model assumptions were inspected using Pearson- and Deviance-residuals as well as DIFBeta and the HAT matrix diagonal [10]. No significant interactions were found, and the model assumptions were found to be adequately met.

A 5% significance level was used throughout. The models were computed with the use of the statistical package SPSS version 18.0.

## Results

Baseline characteristics of smokers and non-smokers are presented in Table 1. The prevalence of men with normal weight was higher among smokers compared to non-smokers, and work capacity was lower.

**Table 1:** Baseline clinical and laboratory variables in 821 healthy men aged 50.7–59.9 years, according to smoking status

	**Non-smokers**	**Smokers**	
	**n = 452**	**n = 369**	**p-value**
Age (years)	55.9 (2.9)	55.8 (2.7)	0.60
BMI (n,%)			
< 25	239 (52.9%)	231 (62.6%)	0.016
25–29.9	196 (43.4%)	124 (33.6%)	
≥ 30	17 (3.8%)	14 (3.8%)	
Total work performed (kJ)	99 (38)	85 (29)	<0.001
SBP (mmHg)	135 (19)	133 (20)	0.20
DBP (mmHg)	93 (10)	92 (11)	0.23
Cholesterol (mmol/l)	6.8 (1.2)	6.8 (1.1)	0.46

When otherwise not specified, results are presented as mean (standard deviation).

During follow-up, 252 of the 821 participants reached the age of 85. Smoking status was strongly and significantly related to longevity in the unadjusted analysis, and as well as after adjusting for age, BMI, physical fitness, cholesterol and blood pressure (Table 2). Among non-smokers, 37.2% reached the age of 85, compared to 17.8% among men smoking at least 10 cigarettes pr day.

**Table 2:** Associations between smoking status and longevity (reaching the age of 85) in 821 initially healthy middle-aged men

**Daily number of cigarettes**	**n**	**Survived 85 yrs**	**Unadjusted**			**Adjusted**		
		**n (%)**	**OR**	**[95% CI]**	**p-value**	**OR**	**[95% CI]**	**p-value**
		**[95% CI]**						
0 (reference)	452	168 (37.2%)	**-**	**-**	**-**	**-**	**-**	**-**
		[32.8–41.7%]						
1–9	156	46 (29.5%)	0.71	[0.48–1.05]	0.084	0.68	[0.45–1.02]	0.060
		[27.7–37.0%]						
≥10	213	38 (17.8%)	0.37	[0.25-0.55]	<0.001	0.39	[0.26-0.58]	<0.001
		[13.1–23.4%]						

Both unadjusted results, and results adjusted for age, BMI, physical fitness, cholesterol and systolic blood pressure, are presented.

Among non-smokers, low BMI and high physical fitness were significantly related to longevity, both with and without adjustment for confounders (Tables 3 and 4). Among men with normal weight, 46.0% reached the age of 85, compared to 28.6% among men who were overweight (BMI between 25 and 29.9). Among men with physical fitness in the highest tertile, 48.8% reached the age of 85, compared to 27.9% in the lowest tertile.

**Table 3:** Longevity (reaching the age of 85) according to smoking status, age, BMI and physical fitness at baseline in 821 initially healthy middle-aged men (unadjusted results)

	**Non-smokers n = 452**					**Smokers n = 369**				
	**N**	**Survived 85**	**OR**	**[95% CI]**	**p-value**	**N**	**Survived 85**	**OR**	**[95% CI]**	**p-value**
		**n (%)**					**n (%)**			
		**[95% CI]**					**[95% CI]**			
Age										
10 years increase			1.09	[0.56–2.13]	p = 0.80			1.93	[0.79–4.69]	p = 0.15
BMI										
<25 (ref. cat)	239	110 (46.0%)				231	56 (24.2%)			
		[39.8–54.4%]					[19.0–30.1%]			
25–29.9	196	56 (28.6%)	0.47	[0.31–0.70]	p < 0.001	124	26 (21.0%)	0.83	[0.49–1.40]	p = 0.49
		[22.6–35.2%]					[14.5–28.8%]			
≥30	17	2 (11.8%)	0.16	[0.04–0.70]	p = 0.015	14	2 (14.3%)	0.52	[0.11–2.40]	p = 0.40
		[2.0–33.7%]					[2.5–39.7%]			
Fitness (tertiles)										
Low (ref. cat.)	140	39 (27.9%)				166	30 (18.1%)			
		[20.9–35.7%]					[12.8–24.5%]			
Medium	150	50 (33.3%)	1.30	[0.78–2.14]	p = 0.31	115	27 (23.5%)	1.39	[0.78–2.50]	p = 0.27
		[26.1–41.2%]					[16.4 31.9%]			
High	162	79 (48.8%)	2.47	[1.52–3.99]	p < 0.001	88	27 (30.7%)	2.01	[1.10–3.66]	p = 0.023
		[41.1–56.4%]					[21.7–40.9%]			

**Table 4:** Longevity (reaching the age of 85) according to smoking status, age, BMI and physical fitness at baseline in 821 initially healthy middle-aged men (adjusted results*)

	**Non-smokers**			**Smokers**		
	**n = 452**			**n = 369**		
	**OR**	**95% CI**	**p-value**	**OR**	**95% CI**	**p-value**
Age, 10 years increase	1.45	0.71–2.93	0.31	2.43	0.96–6.15	0.060
BMI, 25–29.9 vs. <25 kg/m^2^	0.54	0.36–0.82	0.004	0.92	0.53–1.59	0.76
BMI, ≥30 vs. <25 kg/m^2^	0.21	0.05–0.96	0.045	0.84	0.17–4.12	0.83
Fitness, medium vs. low	1.18	0.71–1.98	0.52	1.31	0.72–2.39	0.38
Fitness, high vs. low	1.88	1.12–3.13	0.016	1.80	0.95–3.39	0.070
Cholesterol (1 SD increase)	0.84	0.69–1.02	0.083	1.03	0.79–1.33	0.85
SBP (1 SD increase)	0.89	0.73-1.08	0.24	0.83	0.65-1.06	0.14
Daily number of cigarettes,						
≥10 vs. <1-9	-	-	-	0.56	0.34-0.93	0.024

*Each of the variables was adjusted for the other variables presented in the table.

Among smokers, BMI was not significantly associated with longevity. High physical fitness was significantly associated with longevity in the unadjusted analysis, but not after adjustment (Tables 3 and 4).

Blood pressure and cholesterol level were not significantly associated with longevity among smokers or non-smokers when adjusting for age and life style variables (Table 4).

## Discussion

In our prospective study of healthy middle-aged men, we found that smoking, avoidance of overweight and high or medium physical fitness were strong and independent predictors of longevity. Overweight and physical fitness were significant predictors of longevity in non-smokers, but not in smokers. Further, avoidance of overweight was a predictor of longevity in the medium and high fitness group, but not in the low one. Similarly, the predictive effect of physical fitness was stronger among men with normal weight than among overweight men.

Our findings confirm previous studies showing that cigarette smoking is strongly associated with reduced longevity, and that overweight is associated with decreased longevity in non-smoking men [4-6]. The association between overweight and mortality among non-smokers has recently been demonstrated in several epidemiological studies [11-13]. Also, the lack of association between overweight and longevity in smokers is in line with previous studies on the association between overweight and mortality [11-13].

The importance of physical fitness in midlife is in agreement with previous studies showing that high grip-strength [4] and high physical activity [6] are associated with increased longevity, and the established association between physical fitness and long-term mortality [1-3,7,14].

In our study, blood pressure and cholesterol level were not significantly associated with longevity. This is in contrast to studies on predictors of long-term mortality [1-3]. However, our findings must be interpreted with caution, as the number of participants was much lower in our study.

Smoking is associated with low BMI and high mortality [15]. Controlling statistically for smoking does not fully correct this problem since the adjusted results represent a complex combination of associations between BMI and risk of death. However, limiting the analysis to non-smokers is a suitable means to avoid such potential bias. Therefore, we analyzed smokers and non-smokers separately.

Studies investigating the relationship between lifestyle and longevity will generally be complicated by the fact that some participants might have developed obesity [15], and changed their smoking habits [16] or fitness level [7] during the observation period. These facts warrant caution when interpreting the results.

Strengths of our study include that it is among the longest and most complete follow-up studies of initially healthy middle-aged men. Furthermore, physical fitness was measured in our study, as opposed to similar studies of longevity which typically rely on self-reported physical activity. This is important because self-reported physical activity may underestimate the relationship between physical activity and health outcomes [17].

In most similar studies of overweight, obesity and their health consequences, BMI has generally been calculated from self-reported height and weight [12,18]. Using this type of data carries a risk of error and response biases [19,20]. We eliminated this risk by using physical examinations to measure height and weight.

Increased BMI may be due to both increased muscle mass and increased body fat [21]. As increased muscular strength has been shown to predict lower mortality [22], the estimated negative association between overweight and longevity in our study is probably weaker than the true association between longevity and overweight that is due to increased body fat.

The wide confidence intervals for the association between fitness and longevity illustrate the relatively low test power in our study. Thus, further studies with larger number of subjects reaching 85 years are needed to provide more precise estimates of this association.

## Conclusion

Lifestyle factors in midlife are strongly related to mortality. Our study shows the advantage of avoiding smoking and overweight, as well as being in good physical shape, in the sense of a greater probability of reaching at least 85 years of age. The prevalence of overweight and physical inactivity is increasing in high-income countries. This underlines the importance of informing the general population about the benefits of a healthy lifestyle. Increased probability of longevity may be easier to understand for the general public than reduced mortality rates.

## Abbreviations

BMI: Body mass index.

## Competing interests

The authors declare that they have no competing interests.

## Authors’ contributions

TH and LS analyzed and interpreted data, and worked together to draft the manuscript. JE conceived the study and revised the manuscript critically. All authors read and approved the final manuscript.

## Pre-publication history

The pre-publication history for this paper can be accessed here:

http://www.biomedcentral.com/1471-2458/13/831/prepub
